# Formation and Physical Stability of *Zanthoxylum bungeanum* Essential Oil Based Nanoemulsions Co-Stabilized with Tea Saponin and Synthetic Surfactant

**DOI:** 10.3390/molecules26247464

**Published:** 2021-12-09

**Authors:** Liya Zeng, Yongchang Liu, Zhihui Yuan, Zhe Wang

**Affiliations:** 1 Key Laboratory of Comprehensive Utilization of Advantage Plants Resources in Hunan South, College of Chemistry and Bioengineering, Hunan University of Science and Engineering, Yongzhou 425199, China; liyazeng@nwsuaf.edu.cn (L.Z.); liuyongchang2017@huse.edu.cn (Y.L.); 2 College of Urban and Rural Construction, Shaoyang University, Shaoyang 422099, China

**Keywords:** tea saponin, *Zanthoxylum bungeanum*, biosurfactants, Ostwald ripening, electrostatic repulsion

## Abstract

The purpose of this work was to evaluate the possibility of adding tea saponin (TS) to reduce the synthetic surfactant concentration, and maintain or improve the shelf stability of nanoemulsions. The *Zanthoxylum bungeanum* essential oil (2.5 wt%) loaded oil-in-water nanoemulsions were co-stabilized by Tween 40 (0.5–2.5 wt%) and TS (0.1–5 wt%). A combination of several analytical techniques, such as dynamic laser scattering, interfacial tension, zeta potential, and transmission electron microscope, were used for the characterization of nanoemulsions. Low levels of TS (0.1–0.5 wt%) with Tween 40 had significant effects on the emulsification, and a nanoemulsion with the smallest droplet diameter of 89.63 ± 0.67 nm was obtained. However, in the presence of high TS concentration (0.5–5 wt%), micelles generated by the non-adsorbed surfactants in the aqueous lead to droplets growth. In addition, the combinations of Tween 40 and TS at the high level (>3.5 wt%) exerted a synergistic effect on stabilizing the nanoemulsions and preventing both Ostwald ripening and coalescence. The negative charged TS endowed the droplets with electrostatic repulsion and steric hinderance appeared to prevent flocculation and coalescence. These results would provide a potential application of natural TS in the preparation and stabilization of nanoemulsions containing essential oil.

## 1. Introduction

Nanoemulsions are transparent or translucent systems mostly dispersing two immiscible phases with surfactants as stabilizers [1]. Due to their small droplet size and narrow distribution, nanoemulsions have advantages of reasonable surfactant concentration, large surface area, and high biological properties [1,2]. Furthermore, nanoemulsions can be easily fabricated from food-grade ingredients using relatively simple processes such as mixing and/or homogenization at ambient temperature. However, nanoemulsions are thermodynamically unstable, which means nano-droplets can undergo different destabilization processes with aging time. For instance, coalescence and Ostwald ripening are well-known destabilization mechanisms in nanoemulsions containing essential oils [3,4].

Surfactants are particular ingredients for determining the appropriate approach for the formulation and maintaining the physical stability of nanoemulsions during preparation [5,6,7]. Moreover, surfactants play a significant role in improving the nanoemulsion properties and bioavailability [5,8,9]. At present, many surfactants used in industry are synthetic surfactants, such as Tweens and Spans, have the drawbacks of poor biocompatibility and chronic toxicity. There has been increasing consumer demand for natural, healthy, and sustainable commercial products, which have boosted a trend to replace synthetic surfactants with natural surfactants or natural plant substitutes such as amphiphilic proteins, polysaccharides, phospholipids, and biosurfactants [10,11]. Nonetheless, disadvantages and limitations (e.g., higher working concentration, sensitivity to environmental changes, and high expense) of these natural emulsifiers were also received widespread attention in previous studies [10,11,12,13]. In this case, a combination of natural surfactant and synthetic surfactants to produced fine nano-droplets might be considered as a better method.

Saponins are a kind of biosurfactants consisting of hydrophilic sugar moieties and a hydrophobic steroid or triterpene backbone. These biosurfactants are currently used as foaming agents and stabilizers in beer and soft drinks [14,15]. Some saponins, such as quillaja saponin (QS), have been reported to stabilize the nanoemulsions [16,17]. Although QS has been proved to be an excellent emulsifier, there is interest in developing alternative sources of saponins because of their potential benefits in terms of taste, cost, or sustainability [11]. Tea saponin (TS) is a plant-derived biosurfactant with amphipathic structure, extracted from the by-products of *Camellia* seeds. It has been proved that TS is a safe, nonirritating, and environmentally friendly surfactant with hydrophilic glycosyl and hydrophobic aglycons [18]. Moreover, Zhu, et al. [11] reported that TS is an effective and sustainable source of plant-based emulsifiers that can replace synthetic or animal-based emulsifiers in many commercial applications. Even so, applications of TS in formation and stabilization of essential oil based nanoemulsions are still very limited.

*Zanthoxylum bungeanum* Maximum. essential oil (ZBEO), commonly named Huajiao essential oil, is derived from *Z. bungeanum* Maximum. by hydrodistillation or supercritical fluid CO_2_ extraction. Due to its rich amounts of active compounds such as linalool, limonene, eucalyptol, and sabinene, ZBEO has been widely used as antifungal, antioxidant, and special flavoring agent in food industry [19,20,21]. Despite it is effectiveness, utilization of ZBEO is hampered due to the lack of better formulation. ZBEO has poor water solubility and high volatility, therefore, this study was carried out to develop nanoemulsions containing ZBEO based on the emulsion phase inversion (EPI) method. In general, the formation, stability, and performance of nanoemulsions may be improved by using a combination of two or more different emulsifiers, rather than an individual type [12]. TS has an amphiphilic structure and surface activity, therefore, we hypothesize that the combination of TS and synthetic surfactant could reduce the emulsifier concentration, and maintain or improve the shelf stability of the nanoemulsions. Recently. Ma, et al. [22] fabricated zein/TS nanoparticles that act as the vehicle of lutein, which showed great stability, excellent redispersibility, and high bioaccessibility. Long, et al. [23] evaluated the ability of TS to form and stabilize nanosuspensions and investigated the feasibility of TS as a cryoprotectant of hesperidin solid nanocrystals. Yuan, et al. [24] produced the pH-driven binary composite nanoparticles based on zein and TS without any organic reagents and high-energy equipment. Nevertheless, to the best of our knowledge, the emulsifying property and physical stability of ZBEO based nanoemulsions using TS with synthetic surfactants have not been investigated and discussed. Therefore, the primary purpose of this study was to study the formation and stability properties of ZBEO nanoemulsions using the combinations of synthetic surfactant and TS. Firstly, we investigated the chemical components of ZBEO. Furthermore, the effect of synthetic surfactants and TS on the formation of nanoemulsions were investigated,. Then, the properties of droplets, zeta potential, interfacial tension, and microstructure were studied to explore the feasibility of replacing synthetic surfactant with TS. Further, we analyzed and compared the shelf stability of ZBEO nanoemulsions in the absence or presence of TS.

## 2. Results and Discussion

### 2.1. ZBEO Chemical Compositions

Previous studies suggested that oil phase compositions and physical properties had an appreciable effect on nanoemulsion formation and stabilization [4,25,26]. In the first step, chemical components analysis of ZBEO were performed by GC-MS. As shown in Table 1, different monoterpenes, sesquiterpenes, and fatty acids were identified. The predominant compounds were D-limonene (13.42%), linalyl acetate (11.25%), and linalool (8.94%) in ZBEO obtained by supercritical CO_2_ fluid extraction. Other major compounds were β-pinene (6.18%), palmitic acid (6.89%), 9,12-octadecadienoic acid (Z,Z)-(6.42%), and oleic acid, (Z)-(7.68%). In previous studies, the predominance of the same compounds was reported but with differences in the quantitative profile [21,27,28]. Moreover, ZBEO was also found to contain various saturated and unsaturated fatty acids [20]. The chemical compositions and proportions of EO is highly influenced by the cultivar, geographic region, plant tissue, and extraction method [21,29].

### 2.2. Preparation of ZBEO Nanoemulsions

#### 2.2.1. Effect of Surfactant Type

**Figure 1:**
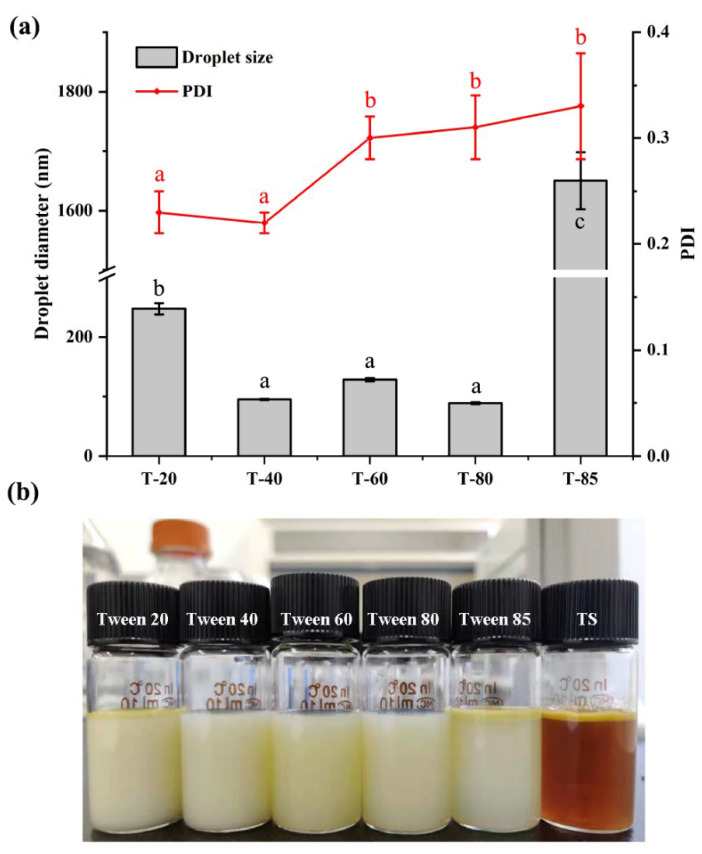
(**a**) Effect of synthetic surfactant and TS on the average droplet size and PDI of ZBEO based nanoemulsions prepared by EPI method. (**b**) Appearance of ZBEO emulsions after storage over night at ambient temperature. ZBEO emulsions were produced using the same oil phase concentration (5 wt%) with different surfactant types (surfactant-to-oil ratio (SOR) of 1:1). ^abc^, means within the same column followed by the same letter are not significantly different (*p* > 0.05) in one-way analysis of variance and Duncan’s test.

To evaluate the influence of synthetic non-ionic surfactants on the formation of ZBEO based nanoemulsions by the EPI method, different combinations of samples were formulated keeping oil phase concentration at 5 wt% (mass ratio of surfactant: ZBEO = 1:1). Tweens were selected because they have previously been shown to be successful in stabilizing various essential oil nanoemulsions [25,30,31]. The type of synthetic surfactants had an appreciable impact on the average droplet size and size distribution of nanoemulsions (Figure 1a, the original data is in the Supplementary Materials). The smallest average droplet size (d = 88.59 ± 1.65 nm, PDI = 0.31 ± 0.03) was obtained in the formulation prepared by Tween 80. The narrowest size distribution (d = 94.90 ± 0.94 nm, PDI = 0.22 ± 0.01) was produced using Tween 40, but the droplets formed were larger than those produced using Tween 80. The largest droplets (d = 127.89 ± 2.49 nm, PDI = 0.30 ± 0.03) were formed when system was stabilized by Tween 60. It should be noted that the emulsion stabilized by Tween 85 and Tween 20 was milky in appearance and oiling off was observed after 12 h of storage at ambient temperature (Figure 1b). There was no strong relationship between droplet size and surfactant hydrophilic–lipophilic balance (HLB) numbers in this study. However, the formulations tended to become more translucent and bluish as the HLB values came close to 15; for example, Tween 80 (HLB = 15.0), Tween 60 (HLB = 14.9), and Tween 40 (HLB = 15.5) had similar HLB values but gave different droplet sizes. Chang and McClements [31] reported a similar observation that a transparent orange oil nanoemulsion containing fine droplets was formed by isothermal low-energy method. Small molecular surfactants such as Tween 40 are known to rapidly coat the surface of the oil–water interface and decrease interfacial tension during emulsification. Moreover, some studies have corroborated that the HLB value required for both limonene and pinene nanoemulsion was 15 [32,33]. Conversely, some papers reported that nanoemulsions using the same surfactants were highly unstable [3,34]. This difference may be attributed to the chemical compositions of essential oils, some of which could stabilize the nanoemulsions by inhibiting the Ostwald ripening [31]. Although the major constituents of ZBEO have appreciable water solubility (β-pinene = 6.95 mg/L, eucalyptol = 2397 g/L, β-ocimene = 0.0145 g/L, and linalyl acetate = 0.3 g/L, data from Sigma^®^), fatty acids have been shown to hinder Ostwald ripening due to the compositional ripening effect [3].

We also investigated the emulsifying property of TS for producing nanoemulsions using the EPI method. In comparison with Tweens, TS was ineffective in forming a stable nanosystem, with visible phase separation and oiling off occurring immediately after preparation (Figure 1b). Mayer et al. [35] have demonstrated that some natural surfactants could not be used to form emulsions using the EPI method. TS does not have the physicochemical characteristics required to form the lamer or bicontinuous structure at the inversion point and facilitate the droplet formation [36]. Moreover, due to the good water solubility, TS could only form an opaque homogeneous mixture before the aqueous phase was added into the ZBEO [35].

#### 2.2.2. Effect of Surfactant Concentration

**Figure 2:**
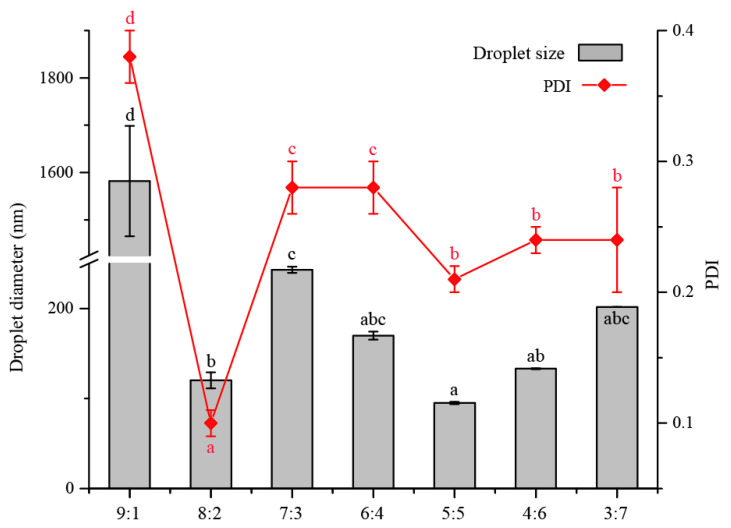
Influence of surfactant-to-oil (SOR) on the average droplet diameter of systems containing 2.5 wt% ZBEO. ^abcd^, means within the same column followed by the same letter are not significantly different (*p* > 0.05) in one-way analysis of variance and Duncan’s test.

Concerning the droplet size and PDI, Tween 40 was selected to investigate the effect of surfactant concentration on the formation of nanoemulsions via changing the mass ratio of surfactant-to-oil (SOR). The overall oil phase of the nanoemulsions was also fixed at 5 wt%, but the mass ratio of Tween 40-to-ZBEO in the oil phase was varied from 9:1 to 1:9. Figure 2 shows the average droplet diameter of emulsions varied between 94.90 nm to 1582.00 nm (emulsions could not form with the SOR at 1:9 and 2:8, data not shown). As expected, the average droplet diameters were clearly dependent on SOR and a minimum droplet size was recorded at SOR = 5:5 (d = 94.90 ± 0.94 nm, PDI = 0.22 ± 0.01). When the droplet size continued to increase with decreasing SOR (<5:5), the prepared emulsions represented oiling off and creaming after 12 h storage. The dynamic scattering results indicated that the emulsions formed at higher SOR (≥6:4) with larger droplet size and transparent or translucent appearance. For example, the largest droplet size and narrowest size distribution were determined when SOR = 9:1 (d = 1582.00 ± 116.75 nm, PDI = 0.38 ± 0.02). Many physicochemical phenomena may account for the changing in droplet size with different surfactant combinations. The decrease in droplet size was attributed to the relatively sufficient surfactant concentration to decrease the oil–water interfacial tension, cover the ZBEO droplets, and prevent the droplet coalescence [11,23,37]. On the other hand, the increasing in droplet diameter at higher Tween 40 levels can be attributed to the appearance of micelles, which promoted the depletion flocculation when droplet size was relatively large; however, this effect can be hampered once the droplet dimension below a certain level [7,38]. Based on these results and in connection with the aim of this study, the optimum SOR was 5:5 for further experiments.

### 2.3. Influence of TS on The ZBEO Nanoemulsions Formation

**Figure 3:**
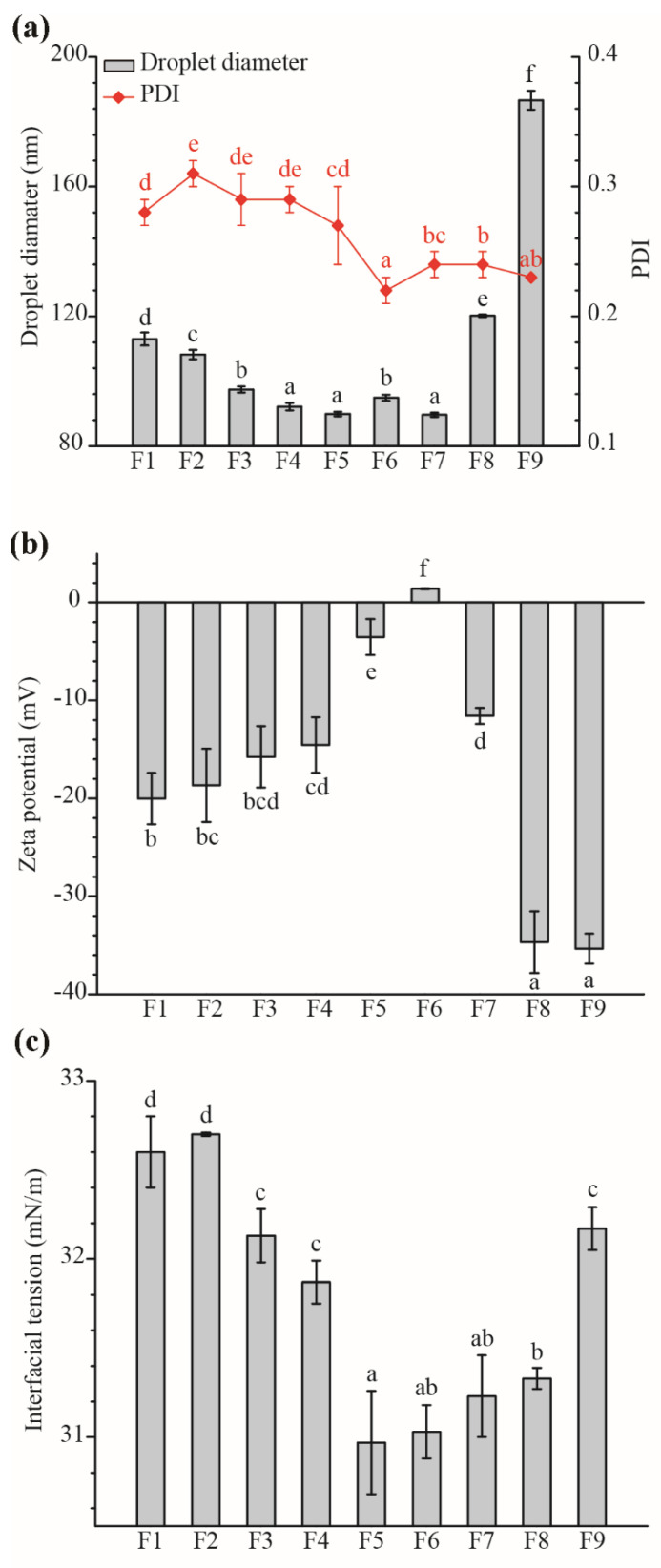
Droplet size (**a**), zeta potential (**b**), and interfacial tension (**c**) of ZBEO nanoemulsions with different ratio of Tween 40 with TS. The data represent the mean ± SD (*n* = 3). ^abcdef^, means within the same column followed by the same letter are not significantly different (*p* > 0.05) in one-way analysis of variance and Duncan’s test.

To evaluate the feasibility of adding TS to reduce the synthetic surfactant concentration, the emulsifying properties of TS/Tween 40 combinations were evaluated. The total ZBEO content in the final nanoemulsions was kept at 2.5 wt%, while the surfactant concentration was varied by altering the amount of Tween 40/TS. As shown in Table 2 and Figure 3, TS had a significant impact on the droplet size, zeta potential, and interfacial tension of ZBEO nanoemulsions. Droplets in Tween 40 nanoemulsion were small in size with uniform shapes (d = 94.90 ± 0.94 nm, PDI = 0.22 ± 0.01). In the presence of a relatively low TS concentration (0.1–0.5 wt%, F4–F5), the droplet size became significantly smaller than that sample (F6) prepared by Tween 40 alone. Nevertheless, there was an obvious increase in the droplet size of the nanoemulsion when the concentration of TS was increased further. Similar trends in droplet size were noted for replacement Tween 40 with TS at a fixed SOR 5:5, with higher TS levels in samples (F7–F10). Furthermore, as the Tween 40 concentration below 0.15 wt%, phase separation and oiling off were observed after preparation for 12 h. These results indicated that Tween 40 had more a efficient emulsifying property than TS. Interestingly, sample F7 showed the expected smallest droplet size and narrower size distribution (d = 89.63 ± 0.67 nm, PDI = 0.24 ± 0.01). Thus, it could be speculated that there was a synergistic effect in ZBEO nanoemulsion co-stabilized by Tween 40 and TS at certain TS concentrations. Similar results in different types of colloid dispersions have been investigated, where TS decreased droplet size at low content and interfered the droplets formation at high content [24]. With incorporation of TS, it adsorbed in the oil droplets surface, thereby stabilizing the oil droplets; thus, it is difficult to reduce the droplet size [10]. Another suggested hypothesis for this observation was regarding the surfactant concentration: as the TS concentration increased, the effective volume fraction of the dispersed phase dropped due to charge screening by more counterions from TS, but depletion attractions generated by micelles in the continuous phase led to extensive droplet aggregation [6].

The electrical property of the droplets in nanoemulsions were characterized in terms of their zeta potential. As shown in Figure 3b, the zeta potential value of F6 was positive, 1.41 ± 0.05 mV. When the concentration of TS was increased from 0.1–5 wt% (F5–F1), the zeta potential values decreased significantly from −3.53 mV to −20.02 mV. In replacement Tween 40 with TS (F6–F9), the zeta potential values were also decreased and the lowest (−35.34 mV) at the 0.5 wt% Tween 40 concentration. These negative charges may be accounted for by the carboxylic acid groups on the absorbed TS chemical molecules [11,22]. Considering that all ZBEO based nanoemulsions with TS presented droplet diameter below 200 nm, we could propose that TS/Tween 40-coated droplets were stabilized by electrostatic repulsion. Nanoemulsions containing Tween 40 alone were mainly stabilized by steric repulsions attributed to the large polymeric head of nonionic/polymeric surfactants, and almost zero potential could be observed [11]. Previously, Riquelme, et al. [17] suggested that zeta potential should less than −30 mV to guarantee a good physical stability of nanoemuslions. Although increasing TS percentage can improve the absolute zeta potential value, physical stability of nanoemulsions should be considered. For example, the zeta potential varied from −34.68 ± 3.16 mV (F8) to −43.60 ± 4.17 mV (F10), phase separation (F8 and F9) and oiling off (F10) were observed immediately after preparation. This suggested that both steric and electrostatic repulsions should be responsible for the physical stability of these nanoemulsions.

Interfacial tension is the surface tension that exists between the oil and aqueous phase, which plays an important role in droplet forming and stability maintaining. One of the roles of surfactant is to lower the resistance to droplet breakup by reducing the interfacial tension [11,39]. Figure 3c shows the variation of interface tension with different Tween 40/TS combinations at room temperature. When there was no TS, the interfacial tension number of ZBEO nanoemlsion was 31.03 mN/m. After the addition of TS, the interfacial tensions of nanoemulsions showed similar values of around 31 mN/m. For example, F5–F1; TS was gradually covering droplets to provide sufficient repulsions as the interfacial tension values were slightly increased from 30.97 to 32.7 mN/m. These measurements of interfacial tension were consistent with the determinations of increasing in droplet size, since higher surface tension prevented the smaller droplets formation. Thus, it could be concluded that 2.5 wt% Tween 40 was a sufficient amount to reduce the interfacial energy and promote the formation of droplets. In most cases, smaller molecular surfactant has the highest capability in forming smaller droplets because of its much lower dynamic interfacial tension. The lower molecular weight of TS (1241 g/mol) and Tween 40 (1277 g/mol) generated a greater translational entropy of surfactant effect opposing adsorption [12]. This is in contrast to other studies reporting that TS has a stronger affinity for the oil–water interface than Tween 80 (1309 g/mol) and QS (1650 g/mol) [11]. This difference in the interfacial property of TS could be explained by the compositions of ZBEO and TS, especially the essential oils always have complex compositions. On the other hand, the surface tension values became constant in the presence of TS, which could be attributed to the saturation of surfactant concentration. Above a particular surfactant concentration, called critical micelle concentration (CMC), the surfactants begin to form self-assembled micelles and surface tension values becomes almost constant [38].

### 2.4. TEM

**Figure 4:**
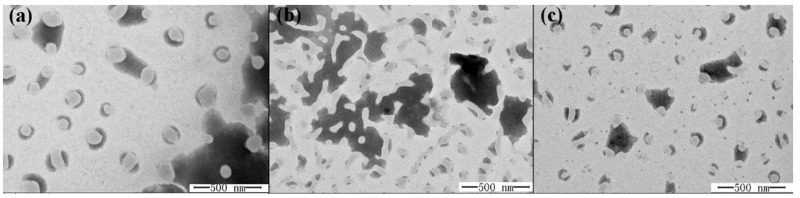
TEM images show nanoemulsions prepared with sample F5 (**a**), F6 (**b**), and F7 (**c**).

Microscopy observations provided a clear understanding of the morphology, microstructure, and stability of nanoemulsions. Figure 4 shows the microstructure observed by TEM for the nanoemulsions with different TS concentration. Droplets with different sizes were characterized by spherical morphology and homogeneous dispersion within the nanonmeter range, which suggested that F5 (Figure 4a) and F7 (Figure 4c) had a high resistance to gravitation separation during a long-term storage. After replacement of Tween 40 with TS, the droplets exhibited a relatively small droplet diameter and existed as uniform spheres that were distributed evenly throughout the dispersed phase, without significant flocculation or coalescence. These droplet sizes were in concordance with the light scattering results obtained in Table 2. In contrast, a network of droplets is shown in Figure 4b. This structure appears to indicate that droplets of ZBEO based nanoemulsion are stabilized by Tween 40, forming a network.

### 2.5. Influence of TS on The ZBEO Nanoemulsions Stability

Ostwald ripening and coalescence are major destabilization mechanisms of nanoemulsions based on essential oils. Droplets grow by Ostwald ripening due to the difference in the chemical potential of droplets at different sizes, whereas coalescence occurs via droplets collision [40]. One can determine if Ostwald ripening is the major destabilization mechanism if there is a linear relationship between the cube of the radius and time. Lifshitz and Slyozov [41], and Wagner [42] proposed a theory (LSW Theory) for predicting the rate of Ostwald ripening (*ω*):

(1) $$\omega \;=\frac{dr^{3}}{dt}=\frac{8DC_{\infty }\gamma V_{m}}{9\rho RT}$$

where *ω* is the rate of Ostwald ripening, *D* is the diffusion coefficient of the disperse phase in the continuous phase, *C_∞_* is the oils bulk phase solubility, *γ* is the interfacial tension, *V_m_* is the oils molar volume, *ρ* is the density of the dispersed phase, *R* is the gas constant, and *T* is the absolute temperature. If coalescence is the driving force for instability, the change of droplet radius with time may follow the following equation [43]:

(2) $$\frac{1}{r^{2}}\;=\;\frac{1}{r_{0}^{2}}-\frac{8\pi }{3\omega t}$$

where *r* is the average droplet radius after time, *r*_0_ is the droplet radius at *t* = 0, and *ω* is the frequency of rupture per unit of surface of the film. Nevertheless, some studies reported that both Ostwald ripening and coalescence can exhibit a linear relation between the cube of average droplet size and time [40,44].

**Figure 5:**
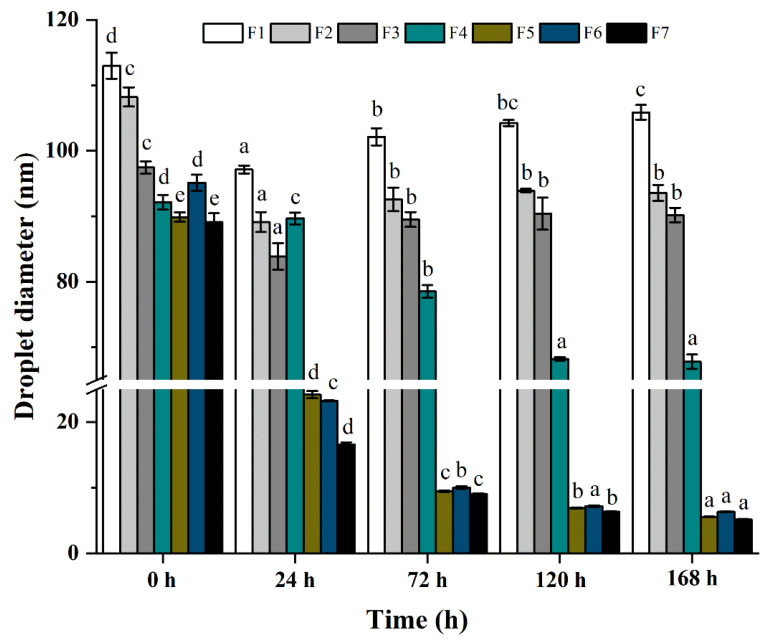
Variations in the average droplet size with time for different samples at room temperature. ^abcde^, different letters indicate significant differences (*p* > 0.05, one-way analysis of variance and Duncan’s test) in the parameters for different times.

In this section, we attempted to underline the effect of TS on the stability of ZBEO nanoemulsions produced via EPI method. In order to investigate the effect of TS on the physical stability of nanoemulsions, droplet size variations in the 168-h storage were demonstrated in Figure 5. All ZBEO nanoemulsions exhibited good stability and depicted falls in droplet size during the 168-h period. For example, the nanoemulsion prepared using Tween 40 alone (F6) showed an obvious decrease in droplet radius (47.45 nm to 11.63 nm) during the first 24 h, and the PDI decreased from 0.22 to 0.20. This may indicate that Ostwald ripening could be the dominant growth mechanism in sample F6. A linear relationship between r^3^ and time can be found in Table 3, when the Ostwald ripening was assumed. In addition, a good correlation of r^−2^ and time was obtained with regression coefficients = 0.99. Although the decrease in the droplet size could be attributed to Ostwald ripening [40], the linear increase in the cube of the average droplet radius is usually taken as the evidence of Ostwald ripening.

In the presence of TS, the decrease in the droplet size of nanoemulsions was also seen and tracked. Figure 5 also shows that the droplet size first decreased and then remained constant as the TS concentration increased from 0 to 5 wt%. For example, there was a significant decrease in the droplet radius of F7 from 44.56 nm to 8.29 nm during the first 24 h, after which the droplet radius remained relatively constant upon further storage. The experimental values of Ostwald ripening and coalescence for TS addition are presented in Table 3. It is noteworthy that r^3^ with time for all samples with Tween 40 and TS combinations are nonlinear within 168 h, whereas, good linear relationships between r^−2^ and time were found when the TS content below 1 wt% (F4–F7). Moreover, values of the coalescence rate showed a decrease with increasing TS concentration, which was consistent with the stability evaluation reported in the previous literatures [4,16]. Thus, taking into account that the growth rate decreases with the increasing surfactant concentration, we might conclude that at low TS levels, coalescence is the major growth mechanism for ZBEO based nanoemulsions. When increasing the concentration of TS, the plot of r^3^ versus time displayed non-linear behavior (F3–F1) and the stability of nanoemulsions was improved. According to the LSW theory, Ostwald ripening could be retarded by the incorporation of a second surfactant with lower interfacial tension. However, as presented in Figure 3c, the interfacial tension value of ZBEO nanoemulsions increased slightly with the addition of TS. Different surfactants have differing absorption kinetics which impacts their ability to prevent droplet coalescence [45]. The cover of TS at the oil interface increased its steric hindrance which possibly reduced the collisions of droplets in the aqueous phase [6]. Thus, it could be concluded that by incorporation into the oil interface and increasing electrostatic interactions between droplets, TS improved the physical stability of the nanoemulsions via retarding the Ostwald ripening.

## 3. Materials and Methods

### 3.1. ZBEO Chemical Compositions

*Zanthoxylumbungeanum* Maxim. (Rutaceae) essential oil (ZBEO, supercritical fluid CO_2_ extraction) was purchased from XuanDe Biological Technology Co. Ltd (Xuande, Guizhou, China) and stored at 4 °C. Tea saponin (TS, 69.53% saponin) was provided by Han Qing Biological Technology Co. Ltd (HanQing, Hunan, China). The synthetic surfactants (Tween 20, 40, 60, 80, and 85) were obtained from Aladdin Co. Ltd. (Alladdin, Shanghai, China). All other chemicals were analytical grade or higher. Deionized distilled water was purified using a Milli-Q gradient system of Millipore (Millipore, Molsheim, France).

### 3.2. GC-MS Analysis of ZBEO

The chemical compositions of ZBEO were analyzed by a Thermo Scientific ISQ 7000 single quadrupole gas chromatography-mass spectrometry (GC-MS, Thermo Finnigan, Waltham, MA, USA) equipped with DB-5 (Agilent, 60 m × 0.25 mm × 0.25 μm) capillary column. The initial temperature was kept at 70 °C for 2 min, increased to 200 °C at a rate of 10 °C/min, and, finally, increased to 280 °C at a rate of 5 °C/min and maintained for 10 min. Samples of 1 μL were injected for analysis with a separation ratio of 15:1 and Helium was used as the carried gas (1 mL/min). Electron impact ionization was generated at 70 eV and the injection and ion source temperatures were 280 °C and 230 °C. The chromatograms were collected by monitoring the total ion currents in *m/z* 30–550. The recorded mass spectra were analyzed using Chromeleon 7.0 (Thermo) software and Wiley-National Institute of Standards and Technology data library.

### 3.3. Nanoemulsion Preparation

Nanoemulsions were prepared using the emulsion phase inversion (EPI) method [46] with some slight modifications. Briefly, the ZBEO-NE were formulated by titrating an aqueous phase (2 mL/min) into an organic phase with constant magnetic stirring (800 rpm, 30 min) at ambient temperature (≈25 °C). The organic phase consisted of different amounts of ZBEO and synthetic surfactant. An aqueous solution of TS was prepared in deionized distilled water and stirred for 30 min. Unless otherwise stated, the water content was 95 wt%.

### 3.4. Droplet Size and Zeta Potential Determination

The average droplet size, size distribution (polydispersity index, PDI), and zeta potential of the ZBEO-NE were determined at 25 °C by using a Brookhaven 90Plus Zeta droplet size analyzer (Brookhaven Instruments Co., Holtsville, New York, NY, USA). A scattering angle of 90° was used at 640 nm. To minimize multiple light scattering effects, samples were diluted appropriately with water before the measurement.

### 3.5. Interfacial Tension Measurement

The interfacial tension (γ) measurement was carried out by a Wilhelmy plate interfacial tensiometer (BYZ-1, Shanghai Hengping Instrument & meter factory, China). The plate was cleaned and dried before each measurement.

### 3.6. Transmission Electron Microscopy (TEM)

The micromorphology of ZBEO nanoemulsion was examined via a TEM instrument (JEM-1200EX, Tokyo, Japan) at 100 kV. Briefly, the nanoemulsions were diluted using deionized water. Then, the samples were added to the copper grids, drying naturally for 20 min at room temperature. Next, copper grids were transferred to TEM instrument for observation.

### 3.7. Stability Evaluation

The physical stability of the ZBEO-NEs was evaluated by measuring the droplet size versus time by the dynamic light scattering at ambient temperature. At each time point, the nanoemulsion was prepared by diluting the original sample 100 times to negate the effects of multiple scattering. The Ostwald ripening rates were obtained from the r^3^ versus time, whereas the coalescence rates were obtained from the r^−2^ versus time.

### 3.8. Statistical Analysis

All statistical tests were repeated at least three times and expressed as the mean ± standard deviation. One-way analysis of variance and Duncan’s test were used to evaluate the statistical significance of difference among samples, and a *p* value of <0.05 was considered statistically significant.

## 4. Conclusions

Based on the results obtained in this work, the combination of TS and Tween 40 enabled the formation of stable nanoemulsions capable of dissolving ZEBO. These nanoemulsions were isotropic and translucent, with an average droplet diameter smaller than 100 nm, and excellent storage stability at ambient temperature. The data clearly showed that the composite surfactants exhibited a synergetic behavior in preparing and stabilizing the ZBEO based nanoemulsions. In the presence of low concentrations of TS, ZBEO nanoemulsons can achieve a smaller droplet size and negative electric charge, which might be attributed to the interfacial properties and electrostatic effects of TS. At higher levels of TS, the droplet size and zeta potential increased significantly while the interfacial tension was almost consistent. Additionally, the main demulsification mechanism for the ZBEO based nanoemulsion was coalescence instead of Ostwald ripening. The electrostatic repulsions ascribing to the carboxylic acid groups on the absorbed TS chemical molecules may be responsible for the remarkable physical stability. Our findings indicate that TS can be used as potentially promising co-emulsifier for nanoemulsions, and may have a good stabilizing ability to delivery essential oil or lipophilic bioactive compounds. Further research is still necessary and important to evaluate the bioavailability, safety, and practical utilization of TS in various fields.

## Figures and Tables

**Table 1:** The chemical compositions (more than 1% of the total oil) identified in ZBEO using GC-MS analysis.

Compounds	Retention Time (min)	Peak Area	Relative Amount (%)
β-Pinene	8.191	107,140,447.22	6.18
D-Limonene	8.987	232,870,450.99	13.42
Eucalyptol	9.062	29,745,305.23	1.71
β-Ocimene	9.113	30,355,650.51	1.75
Linalool	11.966	155,003,077.43	8.94
Linalyl acetate	12.282	195,163,631.66	11.25
Germacrene D	15.772	32,742,265.62	1.89
Palmitelaidic acid	23.190	48,072,287.94	2.77
Palmitic Acid	23.482	119,511,013.73	6.89
9,12-Octadecadienoic acid (Z,Z)-	26.162	111,333,343.35	6.42
Oleic Acid, (Z)-	26.247	133,153,719.97	7.68
β-Eudesmol	31.397	55,400,844.42	4.43

**Table 2:** Formulations of ZBEO-nanoemulsions.

Sample	ZBEO (g)	Tween 40 (g)	TS (g)	Droplet Size (nm)	PDI
F1	0.25	0.25	0.5	112.99 ± 2.00	0.28 ± 0.01
F2	0.25	0.25	0.25	108.21 ± 1.44	0.31 ± 0.01
F3	0.25	0.25	0.1	97.46 ± 0.94	0.29 ± 0.02
F4	0.25	0.25	0.05	92.15 ± 1.14	0.29 ± 0.01
F5	0.25	0.25	0.01	89.88 ± 0.72	0.27 ± 0.03
F6	0.25	0.25	0	94.90 ± 0.94	0.22 ± 0.01
F7	0.25	0.20	0.05	89.63 ± 0.67	0.24 ± 0.01
F8	0.25	0.15	0.10	120.17 ± 0.39	0.24 ± 0.01
F9	0.25	0.10	0.15	186.65 ± 2.92	0.23 ± 0
F10	0.25	0.05	0.20	-	-

**Table 3:** Experimentally-determined Ostwald ripening and coalescence rate for a series of ZBEO nanoemulsions.

Sample	Ostwald Ripening Rate (nm^3^/s)	Correlation (r^2^)	Coalesence (nm^−2^/s)	Correlation (r^2^)
F1	−51.96	−0.30	2.01 × 10^−8^	−0.33
F2	−185.46	−0.05	3.29 × 10^−7^	−0.15
F3	−44.93	−0.28	6.36 × 10^−8^	−0.32
F4	−438.45	0.20	2.69 × 10^−6^	0.92
F5	−373.73	0.21	7.83 × 10^−4^	0.98
F6	−385.91	0.90	6.28 × 10^−4^	0.99
F7	−361.13	0.19	9.00 × 10^−4^	0.99

## Data Availability

Data can be available on request.

## Supplementary Materials

The following are available online. Table S1: The original data of Figure 1. Table S2: the original data of Figure 2. Table S3: The original data of Figure 3. Table S4: The original data of Figure 5.

## Sample Availability

Samples of TS and ZBEO are available from the authors.
